# Exploring gold mineralization in altered ultramafic rocks in south Abu Marawat, Eastern Desert, Egypt

**DOI:** 10.1038/s41598-023-33947-w

**Published:** 2023-05-05

**Authors:** Abdelmonem Eldougdoug, Maha Abdelazeem, Mohamed Gobashy, Mohamed Abdelwahed, Yasser Abd El-Rahman, Ahmed Abdelhalim, Said Said

**Affiliations:** 1 Faculty of Science, Geology Department, Cairo University, Giza, Egypt; 2 Geomagnetism and Geoelectricity Department, National Research Institute of Astronomy and Geophysics, Helwan, Egypt; 3 Faculty of Science, Geophysics Department, Cairo University, Giza, Egypt

**Keywords:** Environmental sciences, Solid Earth sciences

## Abstract

Gold mining is an important strategic sector. The search for mineral reserves is moving deeper as more accessible shallow resources are discovered. Geophysical techniques are now being employed more frequently in mineral exploration because they are quick and can provide crucial subsurface information for discovering potential metal deposits, particularly in high-relief and inaccessible places. The potential for gold in a large-scale gold mining (LSGM) locality in the South Abu Marawat area is investigated using a geological field investigation that includes rock sampling, structural measurements, detailed petrography, reconnaissance geochemistry, and thin section analysis, integrated with various transformation filters of surface magnetic data (analytic signal, normalized source strength, tilt angle), contact occurrence density maps, and tomographic modelling for the subsurface magnetic susceptibilities. The benefits of remote sensing (RS) and its technology in mapping detailed rock differentiation, and characterizing physical objects on the land surface using various spatial, and spectral resolution datasets are integrated. Both aeromagnetic and measured land magnetic profiles are used to investigate the area’s present geological conditions and possible future mining localities. Results indicate that gold mineralization in the study area is linked to the altered ultramafic zones that are associated with faulting and shearing and characterized by a low magnetic susceptibility anomaly.

## Introduction

**Figure 1:**
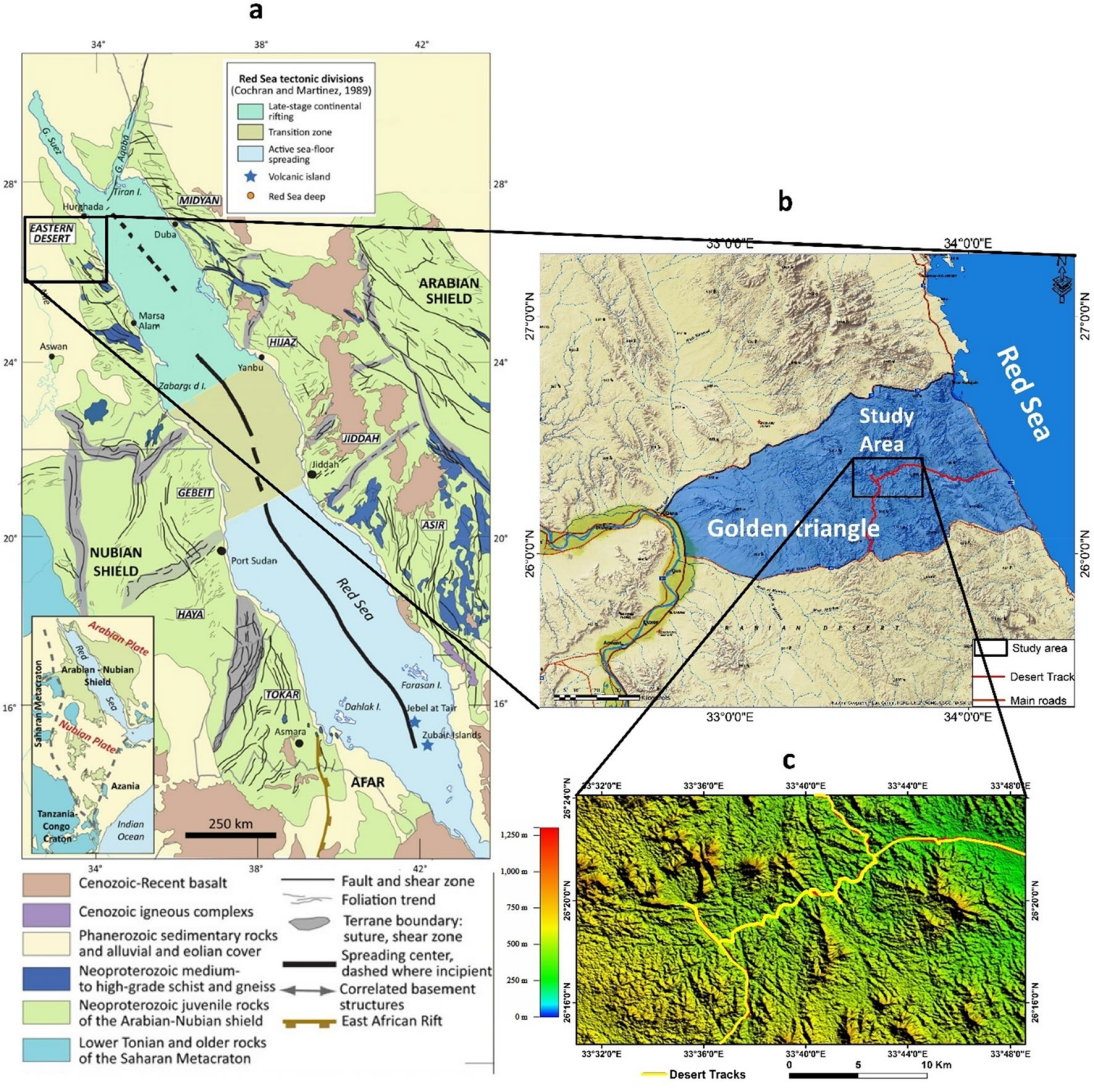
(**a**) Simplified geologic map of the Arabian Nubian Shield (ANS) (modified from^1,7^), (**b**) Location map of the South Abu Marawat area (base map from Esri National Geographic base map 2012^8^), and (**c**) access road and desert tracks as posted on a DEM map (ASTGTM_V3 data of resolution 30 m downloaded from https://earthexplorer.usgs.go,. Produced by ArcGis-10.3 software).

The free economy is greatly affected by gold. Hence, the exploration of gold occurrences and gold deposits attracted the mineral industry all over the world. The Arabian Nubian Shield (ANS) (Fig. 1) is the most potential location for gold occurrences and other mineralization in Northwest Africa and Arabia^1,2^. It occupies both the eastern and western sides of the Red Sea and is regarded as one of the largest surface exposures of Precambrian-era juvenile continental crust. The ANS’s geologic terranes exhibit complexity due to their post-amalgamation occurrences and amalgamation ages that range from 780 to 550 Ma^3–6^. Large portions in many countries, including Saudi Arabia, Egypt, Yemen, Sudan, Eritrea, and Ethiopia, are covered by the ANS. The ANS rocks have a broad range of lithology and geological periods ranging from the Archean (like those in Yemen) to the lowest Palaeozoic, which offers them a strong chance to host many kinds of mineral deposits. The ancient Egyptians made one of the earliest geological attempts to discover and harvest minerals in the world, particularly gold, in the Nubian part of the ANS (in Egypt, Sudan, Eritrea, and Ethiopia) about 1150 BC. The Turin Papyrus of the Wadi Hammamat gold mines and greywacke stone quarries proves such activity^1^. It is dated to the reign of King Ramesses IV (20th Dynasty, 1151–1145 BCE). Similarly, at the Mahd Adh Dhahab gold mine in Saudi Arabia, mining and extraction of precious and basic metals such as gold, silver, copper, zinc, tin, and lead began at least 5000 years ago (Cradle of Gold).

Significant discoveries have been made in the countries covered by the ANS over the past few decades as a result of large and small exploring activities for mineral resources other than hydrocarbons. Understanding the origin and tectonostratigraphic context of the mineral deposits and occurrences in the shield is considerably aided by the numerous researches on the geology and mineral deposits of the ANS.

The Nubian shield (NS), the western limb of the ANS, on the other hand, has a surface area of 100,000 km^2^ and grows along the Red Sea Hills in the Eastern Desert (ED) of Egypt, the southern Sinai, and a few spots in the south Western Desert (Oweinat area)^9^. According to^2^, the NS's basement complex is made up of four main tectonostratigraphic units: (1) high-grade gneisses and migmatites; (2) arc-type volcanic/volcano-sedimentary units with fragmented ophiolites; (3) the Ediacaran Hammamat and Dokhan supra crustal sequences; and (4) granitoid between the Cambrian and Cretaceous.

Before the uplift associated with the split of Arabia and Africa culminated in the Nubian Shield becoming visible as it is now, the rocks of the Nubian Shield were nearly perpetually covered by Phanerozoic sedimentary strata. The majority of the rocks of the Nubian Shield are young Neoproterozoic sediments, which span 1000 km from northern Sudan to northern Egypt. Serpentine or combinations of serpentine, talc, tremolite, magnesite, chlorite, magnetite, and carbonate (talc carbonate schists or soapstone as well as listwaenite) are the main products of the ultramafic rocks associated with NS ophiolites^10^. Ophiolitic ultramafic rocks have been suggested as a source for many gold deposits and gold concentration in the carbonatized varieties of the ophiolitic serpentinites may reach high economic grades ranging from 1 to 10 ppm^11^. According to Buisson and LeBlanc^11^, carbonation of the ophiolitic serpentinite results in magnetite dissolution, the release of gold from its structure, and goals concentration preferably in the carbonatized ultramafic varieties. Thus, the NS is a potential district for gold exploration in the carbonatized ultramafic rocks, and suitable exploration techniques, such as magnetometry that can detect magnetite destruction, are needed to detect the ultramafic rocks as well as their carbonatized varieties which are potential zones for gold exploration.

Fortunately, mineral exploration program workflow is now attracted to geophysical techniques due to their acceptable prices, rapid results, deeper targets, efficiency, and reduced time of exploration and exploitation. Particularly passive aeromagnetic (as a reconnaissance technique) and land magnetic surveys (in detailed quantitative analysis) have been utilized extensively in geologic mapping applications and associated studies. It is employed as an effective technique and has numerous geological uses, such as geologic mapping of potential locations with buried igneous bodies that are frequently linked with mineralization, identifying supra-basement features, and estimating the thickness of sedimentary cover^12^. Moreover, it is also the favourite for studying the tectonic structure, identification of intra-basement faults and uplifts, basin modelling, and structural geometry^13–16^, petroleum exploration modelling of geothermal and groundwater resources^17–19^, allowing the visualization of the geological structure of the upper crust, like faults, aids in the discovery of buried objects such as pipes, drums, and unexploded ordnance^20^. Furthermore, it aids in mineral exploration^13,16,18,21–30^.

The lack of in-depth knowledge on the nature and origin of the gold-bearing formations in the South Abu Marawat region, at the central part of the Golden Triangle (Fig. 1b,c) the most potential gold mineralization zone in the Central Eastern Desert (CED), as well as their connection to existing gold ores and shear zones in the NS, justifies the need for more research. In the current paper, geologic and structural controls on gold mineralization, petrography, and magnetic methods have been employed in a context that would assist in distinguishing the different gold zones of mineralization, altered ophiolitic ultramafic rocks, alteration zones, and direct further exploratory activities in the South Abu Marawat and similar regions. Moreover, structurally, understanding how various types of gold mineralization evolved along the two main trends (NE- and NW-trending tectonic fabrics) in the study area is another main goal of this study together with providing a thorough structural description of the subsurface through the study of the distribution of surface and subsurface magnetic susceptibilities for future gold potentiality. The integration between the geological, geochemical, and aeromagnetic data is done through data overlay in ArcGIS environment. While with ground magnetic data is done by projecting the magnetic profile path on the geological map and then comparing the lithologic contacts and structural lines of the geologic map (together with sample locations and field geologic results) with the ground magnetic stations of the profile. Accordingly, the contacts and faults are placed in their exact position at the top of the profile and then extended through the subsurface as expected from the geological mapping and the distribution of the magnetic susceptibilities resulting from the tomographic inversion of these profiles.

In contrast to the well-known gold quartz veins (QV) connected to diorite/granodiorite intrusion in historic gold mines, this research is intended to learn more about (search for) various types of gold mineralization. The novel idea presented in this research is a workflow for locating new mineralization regions connected to hydrothermally altered ophiolitic ultramafic rocks, utilising a regularised method and integrated geology for subsurface magnetic susceptibility inversion.

### Study area

South Abu Marawat area occupies the central part of the Golden Triangle (Fig. 1a,b), the highly Mineralization potential zone in the eastern desert of Egypt. It is delimited by latitudes; 26° 14' 21.8066" to 26° 24' 06.3516"N and longitudes; 33° 31' 00.6035" to 33° 48' 34.9696" E; and is 528 km^2^ in area. The study area is hardly accessible and occupies the Northern part of the CED (Fig. 1b). Unlike most of the eastern desert, the area is characterized by the lack of ancient gold exploration. Currently, the abundance of gold-bearing rock succession in this region attracts international mining companies to start exploration programs, and hence, gold exploration in that region finds great challenges due to the lack of published literature and detailed studies characterized by scarcity of vegetation cover and deeply incised valleys. These conditions are excellent for utilizing remote sensing and magnetic techniques in geological surface and subsurface mapping and provide pseudo-3D vision which allows field investigation and structural analysis.

## Materials and methods

### Remote sensing data

The processing of multispectral Landsat-8 and ASTER data was used in refining the basement complex of the study area with an emphasis on the ultramafic units by using band combinations and examining band ratios. Previous geological mapping of the area has been reviewed^31–34^ and a new geological map is produced based on satellite image interpretation and field check. Remote sensing has an indirect role in exploration. It is used in lithological mapping, and lithological mapping is then used in exploration.

The preliminary mapping and the detailed rock differentiation are completed based on the studied available previous maps and processing and interpretation of the different satellite images. Landsat-8 and ASTER data used are provided with a maximum resolution of up to 15 m/pixel. This resolution produces an image of a scale that reaches up to 1:40,000 without any remarkable pixilation and is suitable for the present work. Landsat-8 is provided with the Operational Land Imager (OLI) that is differentiated into 9 channels of spatial resolution of 30 meters (visible, NIR, SWIR); and 15 meters for one panchromatic channel rather than two thermal bands of (100 meters) come from the TIRS sensor. The ASTER instruments acquire data in three native spatial resolutions: VNIR (Visible and Near-Infrared) Bands 1, 2, 3N, 3B^1^: 15 meters. SWIR (Shortwave Infrared) Bands 4–9: 30 meter. TIR (Thermal Infrared) Bands 10–14: 90 meter. The following Landsat-8 and ASTER data scenes are used in rock units’ discrimination and mapping Landsat-8: scene ID = LC08_L1TP_174042_20180616_20180703_01_T1, acquired on 2018/06/16, ASTER scene ID = 1-AST_L1T_00303262006083527_20150513171836_58761, acquired on 2006/03/26, and ASTER scene ID = 1-AST_L1T_00303262006083536_20150513171833_ 56433, acquired on 2006/03/26.

Landsat-8 and ASTER data are downloaded from https://earthexplorer.usgs.gov. The different processing techniques are applied to the images using Envi-5.3 software.

Moreover, different remote sensing techniques are applied to construct the base maps of the selected areas. Processing of satellite data has been accomplished through the following techniques: (1) Layer Stacking of the different bands in the image, (2) Mosaicking of images, where it is needed in the ASTER data, (3) Sub-setting of images to get the exact areas needed, (4) Construction of False Colour Composite images (FCC) using the band combination and band rationing techniques to obtain the best lithologic discrimination and identifying the ultramafic bodies, and (5) image enhancement and HSV merging techniques are used during the processing. Additionally, the UTM projection (WGS84 datum, zone 36N) is utilized for the produced images.

In general, several band ratios studied by many authors are tested for best rock differentiation as shown in Table 1**.** The band combinations 7, 5, 3 and 7, 3, 1 in RGB for Landsat 8 and ASTER data, respectively, are used in the preliminary base mapping. The band ratios 6/7, 6/2, 4/2 and 6/8, 4/6, and 4/5 in RGB for Landsat 8 and ASTER data, respectively, (modified after Refs.^35,36^) were found to be the most efficient composite colour band ratios in rock differentiation, particularly the ultramafic bodies and are used in detailed geological mapping.

**Table 1:** Examined band ratios.

Band ratio	Source	Examination
8/6, 8/7, 4/7, ASTER	^37^	Excellent
9*/*5, 1*/*3, 6*/*3, ASTER	^37^	Excellent
7/5, 3/1, 4/3, ETM Landsat	Hydrothermal band ratio	Excellent
5/7, 5/4, 3/1, ETM Landsat	Mineral composite band ratio	Good
4/1, 3/1, 12/14, ASTER	^38^	Good
5/7, 5/1, 4/3, TM Landsat	^39^	Good
4/3, 3/1, 5/7, TM Landsat	^39^	Good
7/6, 6/5, 6/4, ASTER	^40^	Good
4/3, 5/4, 5/7, ETM Landsat	^41^	Fair
4/5, 5/6, 7/6, ETM Landsat	^42^	Fair

It is well known that the band combinations 7, 5, 3 and 7, 3, 1 in RGB for Landsat 8 and ASTER data represent the best band ratios for general lithologic differentiation. The ASTER band ratio 6/8, 4/6, and 4/5 used in the present work is modified after^36^. In this work, the authors used the ratio 6/8, 4/7 and 4/5. This detailed stud indicates that:

- The ASTER band ratio (6/8) shows a contrast between Fe-rich rocks of mafic–ultramafic affinity, arc metavolcanics, gabbro and diorite, and the granitoid intrusions.
- The ASTER band ratio (4/7) is useful in identifying the serpentinites and talc-carbonate-rich rocks
- the ASTER band ratio (4/5) is useful in discriminating gabbro and diorite rocks.

Accordingly, we used the same ratios with the modification of 4/7 to 4/6 and an RGB band ratio image is constructed which showed better results.

For Landsat 8 OLI, band ratios of 4/2, 6/7, and 6/5 in RGB are essential in discriminating lithological units, altered rocks, and vegetation^43^. This ratio is known as the Sabins ratio^44^, which is widely used by many authors to highlight mafic and ultramafic rock units (e.g. Refs.^45,46^. The reference^43^, indicated that:

1. Band ratio 4/2 was used to highlight iron oxide.
2. Band ratio 6/5 was used to highlight ferrous minerals (iron-bearing) other than iron oxides such as amphiboles, olivine, and pyroxene. This is because these minerals tend to have high adsorption in band 6 and reflectance in band 5.
3. Band ratio 6/7 was used to highlight hydroxyl-bearing rocks.

It is found that this ratio is suitable for our target of mapping to highlight the mafic and ultramafic rocks. In the present work, the ratio 6/5 is modified to 6/2 which gives better discrimination in the RGB image. The band combination and band ratio images are used as base layers in the ArcGIS database to trace the lithologic contacts and structures on the geologic map accurately based on the visual interpretation of the differentiated units.

## Field geology

Structurally, the CED is a characteristic domain in the ANS^47^. It is dominated by NW-SE structural elements that extend northward to the Northern Eastern Desert and truncated southward by the E-W structural domain of the El-Barramyia—Wadi Mubarak belt (e.g.: Ref.^48^). High-strain shear zones are an important structural element in the architecture of the CED. They extend for several kilometres across the Precambrian basement and represent major crustal boundaries between different structural domains. The basement rocks of the CED include high to medium-grade gneiss and amphibolite complex, greenschist ophiolitic and island-arc rocks, and syn-late tectonic calk-alkaline granites^49^. This rock assemblage was developed during the Late Neoproterozoic Pan-African orogeny^49–56^. ANS was developed in three tectonic–magmatic stages; island-arc stage, orogenic stage, and cratonization stage^57^.

A detailed site investigation in South Abu Marawat has been conducted to verify remote sensing mapping of the rock units and collection of the rock samples and structural measurements. Three traverses were followed during the field investigation selected based on the preliminary mapping of the rock units and main structural elements identified in different remote sensing analyses and the reconnaissance fieldwork. Because of the difficult accessibility of the area, the fieldwork and sampling are focused on the ultramafic rocks, alteration zones, and their contacts with other rock units.

### Magnetic data

To achieve the objectives of this work, a multidisciplinary magnetometry approach including airborne and ground magnetic measurements is used with a variety of filtering and subsurface modelling. Results are then integrated with geological interpretation. The Egyptian General Petroleum Corporation’s total magnetic intensity map is subjected to several magnetic filters^58^. The survey parameters^59–61^ are magnetometer: Varian VIW 2321G4 Single-Cell Caesium Vapor; altitude 120 m (394 feet); Terrain Clearance, Average total Magnetic field intensity: 42, 425 gammas; Flight line spacing: Traverse = 1.0 KMS, Tie = 10.0 KMS; Flight line direction: Traverse = 45_/225, Tie = 135_/315_; Declination: 1.9° East, and Inclination: 32.8° North. Moreover, a total of 165 land magnetic stations were measured using a portable Geometrix G-857 proton precession magnetometer (station spacing ranges between 20 to 50 m) along three long selected profiles crossing the main geologic units for detailed inversion and 2D modelling. The data were then corrected for diurnal variation using base station (BS) magnetometer data. The BS magnetometer is a G-856 unit calibrated with the portable unit and used for recording at regular time intervals for automatic monitoring and recording diurnal variations in the earth's magnetic field every 120 s. The diurnal correction is performed using a developed MATLAB code to remove its effects from the measured data. Then a simple filter is applied to remove cultural noise and micropulsations. The aeromagnetic survey was used for regional and reconnaissance studies of the area.

The reduced-to-pole (RTP) map is used to minimize the impact of the geomagnetic field’s skewness and then processed using different filters to enhance the signal and extract subsurface controlling parameters. These filters are (a) The Derivative filter to extract lineaments and magnetic contacts, (b) the analytic signal, tilt, and Euler deconvolution filters to explore rock units’ edges and magnetic contacts and depths to magnetic sources^18,30,36,62–64^, (c) the magnetic gradient tensor’s normalized source strength (NSS) filter that serves as an efficient edge detection filter. The NSS filter, in particular, is only slightly and not strongly dependent on magnetization direction for a sizeable class of sources. It is proportional to the strength of the source and reaches its maximum directly above the magnetic source. When looking for mineralization zones associated with magnetic contacts, this technique is crucial in mineral prospecting^18,65^, and (d) the Centre for Exploration Targeting (CET) grid analysis technique examines an image's texture to identify areas of structural complexity and, as a result, assess the likelihood of the existence of a gold deposit^66,67^. This method first locates magnetic discontinuities, then identifies areas of discontinuity, and then analyses structural relationships to detect crossings, junctions or contacts, and changes in direction in the strike. It makes it easier to select the locations that are probably promising^66,67^. Consequently, the method entails three steps: texture analysis, texture ridge detection, and thinning of texture ridges. In this technique, Utilizing texture analysis, the local area of each picture point is described. The standard deviation (STD) filter displays the range of greyscale pixel intensities. The texture ridge detection method is then used to find the laterally continuous ridges in the texture analysis result. Line segment vectorization examines the texture ridges identified by phase symmetry to obtain line segments that are representative of the texture ridges. To do this, a binary (i.e. black and white) grid is created by thresholding the phase symmetry output, designating the texture ridges in the foreground as white and the background as black, and finally magnetic tomographic modelling (e). In this process, the 2-dimensional earth model is assumed to be made up of several prismatic cells, each having an unknown magnetic susceptibility value, and tomographic inversion is carried out under this assumption. A hybrid method that combines targeted regularization with the Marquardt algorithm has been applied. For such a technique, Zondgm2d software is employed. The tomographic section that results shows how the subsurface magnetic susceptibilities may be distributed along the chosen profiles. The centre straight profiles that best fit each modelled profile individually are projected onto all modelled profiles. To get the data ready for inversion along a regular array of data, this method is required. Due to the wide variations in magnetic susceptibility magnitudes, which impede unifying the scale, each profile has its colour scale. Blue colours are used to indicate low susceptibility ratings, whereas red and pink colours indicate high values. Using a KT-20 magnetic susceptibility meter, surface magnetic susceptibilities are also measured along all profiles. The inverted profiles are constrained by the observed data, which are employed as a *prior* information. Such distribution of subsurface magnetic susceptibilities is correlated with known gold occurrences and geologic conditions. Based on this new attribute, new possible potential zones are suggested.

## Results and discussion

**Figure 2:**
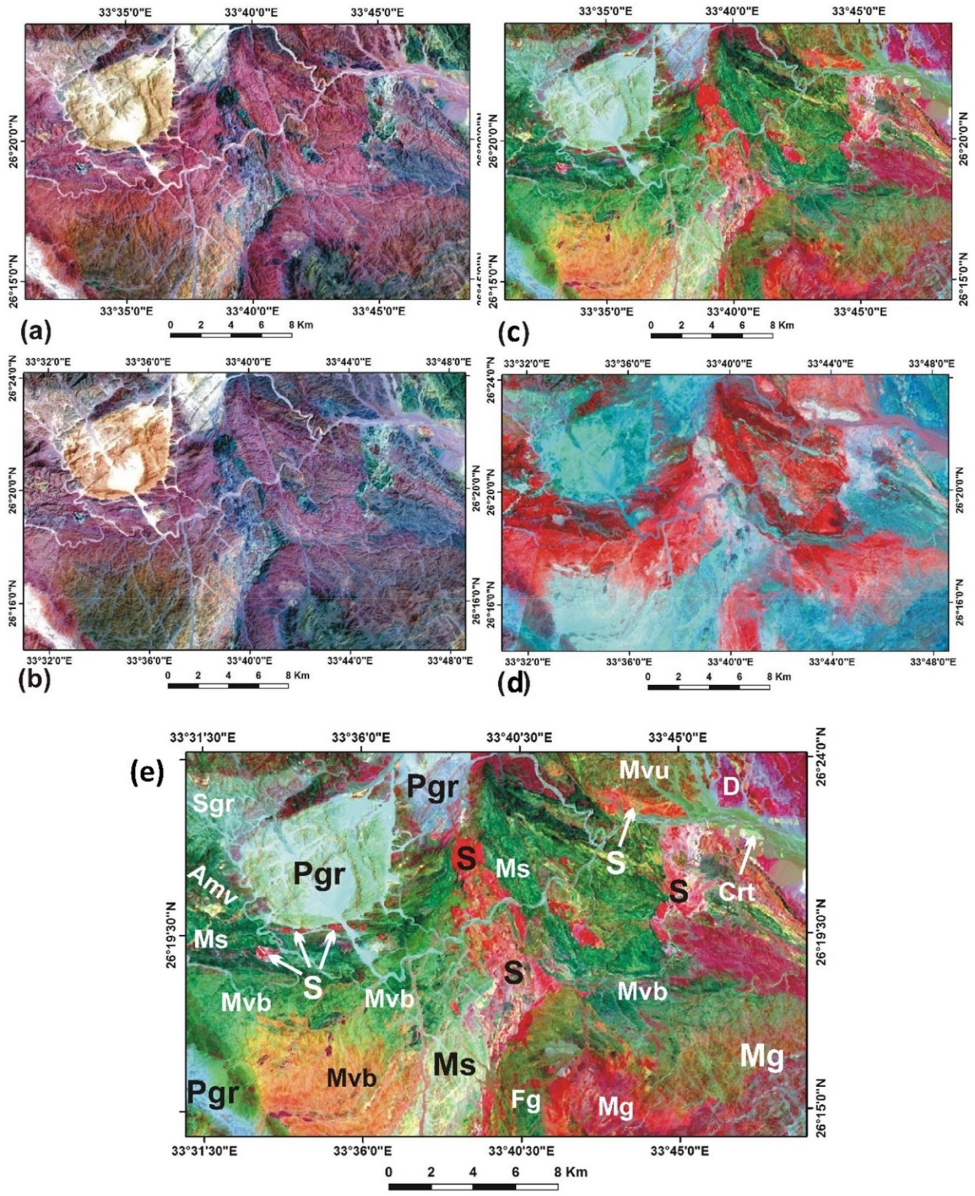
(**a**) Landsat-8 image of South Abu Marawat area of band combination 7,5,3 for RGB., (**b**) ASTER image of band combination 7,3,1 for RGB, both show serpentinites in dark green colour, (**c**) Landsat-8 image of band ratio 6/7, 6/2, 4/2 in RGB, (**d**) ASTER image of band ratio 6/8, 4/6, 4/5 in RGB, and (**e**) Landsat-8 image of band ratio 7/6, 6/2, 4/2 for RGB showing the distribution of rock units. *S* Serpentinite, *Mg* Metagabbro, *Mvb* Basic metavolcanics, *Mvu* Undifferentiated, metavolcanics, *Amv* intermediate to acidic metavolcanics, *Ms* Metasediments, *Sgr* Syn-orogenic granite, *Fg* Fresh Gabbro, *Pgr* post-orogenic granite, *D* Dokhan volcanics, Crt Cretaceous sediments. Landsat-8 and ASTER data are downloaded from https://earthexplorer.usgs.gov. Images are processed by Envi-5.3 software.

The remote sensing is based on the band combination images 7, 5, 3, and 7, 3, 1 of the Landsat-8 and ASTER data, respectively, as shown in Fig. 2a,b. Both images did not show any reliable difference and both were used for constructing the base map. For more detailed discrimination of the different rock units, the mathematical band ratios of 6/7, 6/2, 4/2, and 6/8, 4/6, and 4/5 in RGB, for Landsat 8 and ASTER data, respectively, represented the most efficient composite colour (Fig. 2c,d). The band ratio images (Fig. 2c,d) show a distinct variation in the spectral reflectance of the different lithological units, particularly the ultramafic bodies, that resulted in a more detailed distribution of rock units and structural trends than previous geological mapping. The rock units are interpreted and classified into 11 units according to their characteristic reflectance in the satellite images, geological setting, and petrography as shown in Fig. 2e. The serpentinite appears in bright red colour, the metagabbro in dark red, the basic metavolcanics generally in green, the undifferentiated metavolcanics in dark greenish red, the intermediate to acidic metavolcanics in very dark green, the metasediments in pale blueish green and dark green, the Syn-orogenic granite in reddish blue, the fresh gabbro in dark reddish green, the post-orogenic granite in pale blue, the Dokhan volcanics reddish violet, and the Cretaceous sediments in pale yellowish green.

Several small ultramafic bodies (bright red colour S symbol) located to the south of the rounded post-orogenic granite body (Pgr) at the NW part of the area can be distinguished.

**Figure 3:**
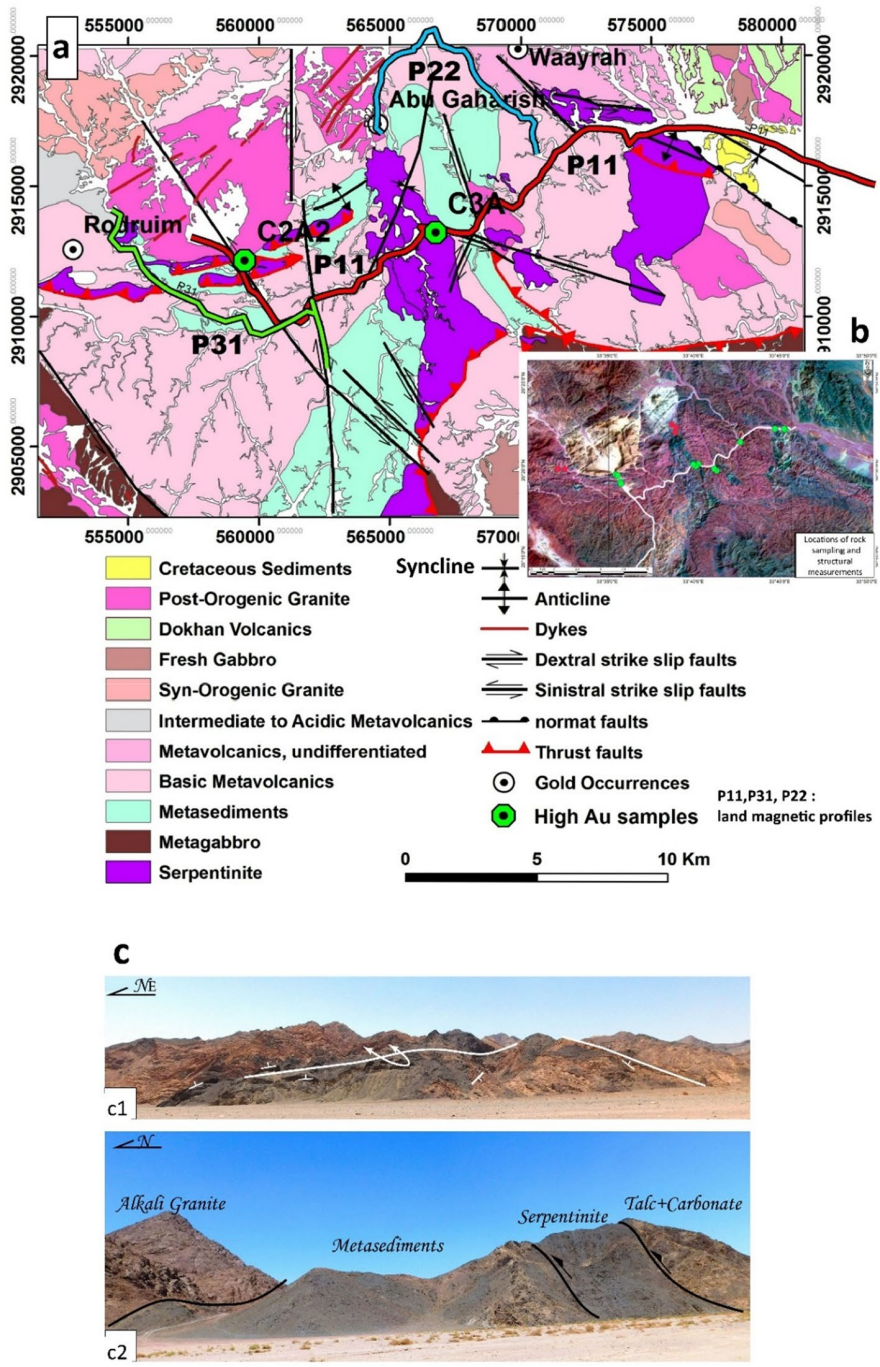
(**a**) Geological map of the South Abu Marawat area constructed by ArcGis-10.3 (https://arcgis.software.informer.com/10.3/), (**b**) Field stations plotted on a False colour composite of Landsat-8 of band combination 7,5,3 for RGB processed by Envi-5.3 (https://www.envi.com/), (**c**(1)) Foliation and folding of the ultramafic rocks in the eastern part of the study area, and (**c**(2)). Ultramafic thrust sheet is thrust over metasediments, which are intruded by alkali granite.

Consequently, the geological interpretation of satellite images was supported by field documentation of 18 reference field stations. The final geologic map is presented in Fig. 3a. Lithologically, the mapped rock units in the area belong to two lithotectonic sequences; the syn-orogenic sequence including ophiolitic mélange and island arc metavolcanics which is intruded by syn-orogenic granite; the post-orogenic sequence including younger gabbro, post-orogenic granite, and Dokhan volcanic, and overlain by the Phanerozoic sedimentary sequence (Fig. 3b). The ophiolite rock succession is the oldest in the area and is mapped as dismembered units in the central, eastern, and north-western parts of the area.

Ophiolitic rocks are represented by altered ultramafic rocks (serpentinite, and talc carbonate rocks), metagabbro, and metasedimentary matrix rocks. Island arc volcano-sedimentary sequence is widely distributed, wrapped in the ophiolitic rocks, and represented by Calc-alkaline basic and intermediate metavolcanic. The Syn-orogenic sequence was introduced by granodioritic rocks (syn-orogenic granite). The phanerozoic rock succession is exposed in the northeastern parts, faulted down towards the east by NW-oriented normal faults (Fig. 3b).

Structurally, the rock succession of the South Abu Marawat area belongs to the low metamorphosed greenschist facies which exhibit well-exposed foliation and associated folds. Three folds of NE, ENE, and NW orientations were recognized in the metamorphic ophiolites and island arc volcano-sedimentary succession (Fig. 3b). Large scale-NE-oriented fold is mapped in the central part of the area. This fold is cored by serpentinite and island arc-volcanic and flanked by the metasediments. The southern and the northern limbs of this fold are marked by SE to S verging thrust faults (Fig.3b). Minor overturned folds are recognized within the serpentinites in the north-eastern parts of the study area (Fig. 3c).

Thrust faults have E-W to WNW and NE orientations and are represented by thrust sheets that mark the tectonic boundary between the ophiolitic mélange and island arc succession (Fig. 3c). In the northern part of the study area, the ophiolitic mélange is thrust over metasediments along several E-W minor thrust faults (Fig. 3c(2)).

**Figure 4:**
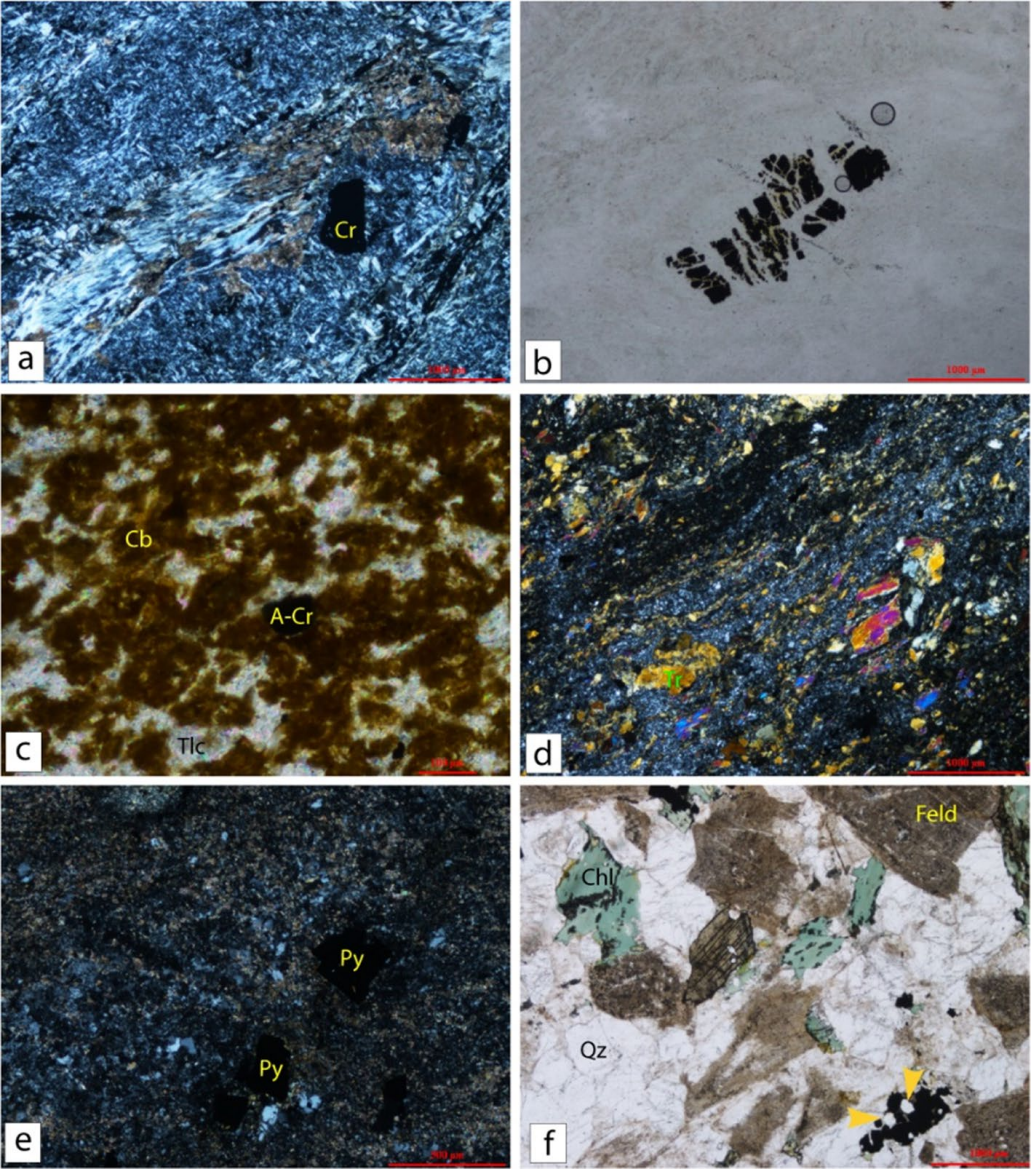
(**a**) Chromite (Cr) crystal in weakly carbonatized serpentinite (XPL). (**b**) Brecciated chromite in sheared serpentinite (PPL) (**c**) Altered chromite (Cr) crystal in stained carbonate (Cb) associated with talc (Tlc) (XPL). (**d**) Oriented tremolite (Tr) in a sheared silica-rich matrix of metamorphosed metavolcanics (XPL). (**e**) Pyrite disseminations (Py) in quartz-sericite-chlorite alteration assemblage (XPL). (**f**) Quartz (Qz), kaolinized feldspar (Feld), chloritized biotite (Chl) and accessory titanite (Ti) and apatite (yellow arrows) are associated with opaque crystals of the Gaharish granite (PPL). (*PPL* Plabe polarized light and *XPL* Crossed polarized light). Thin sections are prepared from samples collected during field trips and investigated using a Nikon Optiphot-Poll polarized microscope equipped with a fully automatic microphotographic unit (Nikon FX32) at the Geology Department, Cairo University (it is too old, and not added to the URL link of the manufacturer).

The ultramafic rocks, which constitute the main blocks of the ophiolitic mélange, are intensively serpentinized; only primary chromite relics are preserved (Fig. 4a). Chromite is brecciated in the sheared serpentinite (Fig. 4b), which is finally altered to talc-carbonate along thrust planes (Figs. 3c and 4).

Due to the complex structural history of the area, the island arc metavolcanic rocks of the South Abu Marawat area are variably deformed (Fig. 4d). They are also variably altered into quartz-sericite-chlorite assemblages, which enclose pyrite disseminations (Fig. 4e). Unlike the ophiolitic and island arc assemblages, the post-orogenic assemblages, which are represented mainly by granitic plutons lack any indication of deformation and were not affected by regional metamorphism (Fig. 4f).

Generally, the area is affected by two right and left-lateral strike-slip fault systems of N-S and WNW orientations, respectively (Fig. 3). The N-S faults are present in the middle part of the mapped area as large faults extended for over 15 km and show the right-lateral offset of the rock contacts. The WNW-oriented faults sinistrally offset-thrust contacts. The eastern part of the study area is marked by a WNW-oriented syncline affecting the Cretaceous Nubian sandstone. This syncline has been developed as drag along the WNW-oriented normal fault of the NNE downthrown side related to the Red Sea tectonics. The structural framework of the South Abu Marawat area is the result of three deformational phases. The oldest is a compressional ductile deformation represented by folds, thrust faults, schistosity, and mylonitic foliation. The second is a shear brittle deformation characterized by dextral and sinistral strike-slip faulting, while the third is an extensional brittle deformation represented by normal faulting resulting in the juxtaposition of Cretaceous sediments against the basement rocks.

Geochemically, three representative samples were analysed by fire assay for their gold content (Fig. 2). The analyses were conducted in the central laboratories of the Egyptian Mineral Resource (EMRA). The analysed samples (Fig. 4) comprise two talc-carbonate or soapstone samples and carbonatized volcaniclastic sample. Thin sections are prepared from samples collected during field trips and investigated using a Nikon Optiphot-Poll polarized microscope equipped with a fully automatic microphotographic unit (Nikon FX32) at the Geology Department, Cairo University. Major and trace element compositions and gold analyses of representative samples from the area are analysed. Gold was detected in the soapstone samples **C2A2** and **C3A** are 1.7-2.2 ppm respectively, while the carbonatized volcaniclastic sample **R2** is barren.

**Figure 5:**
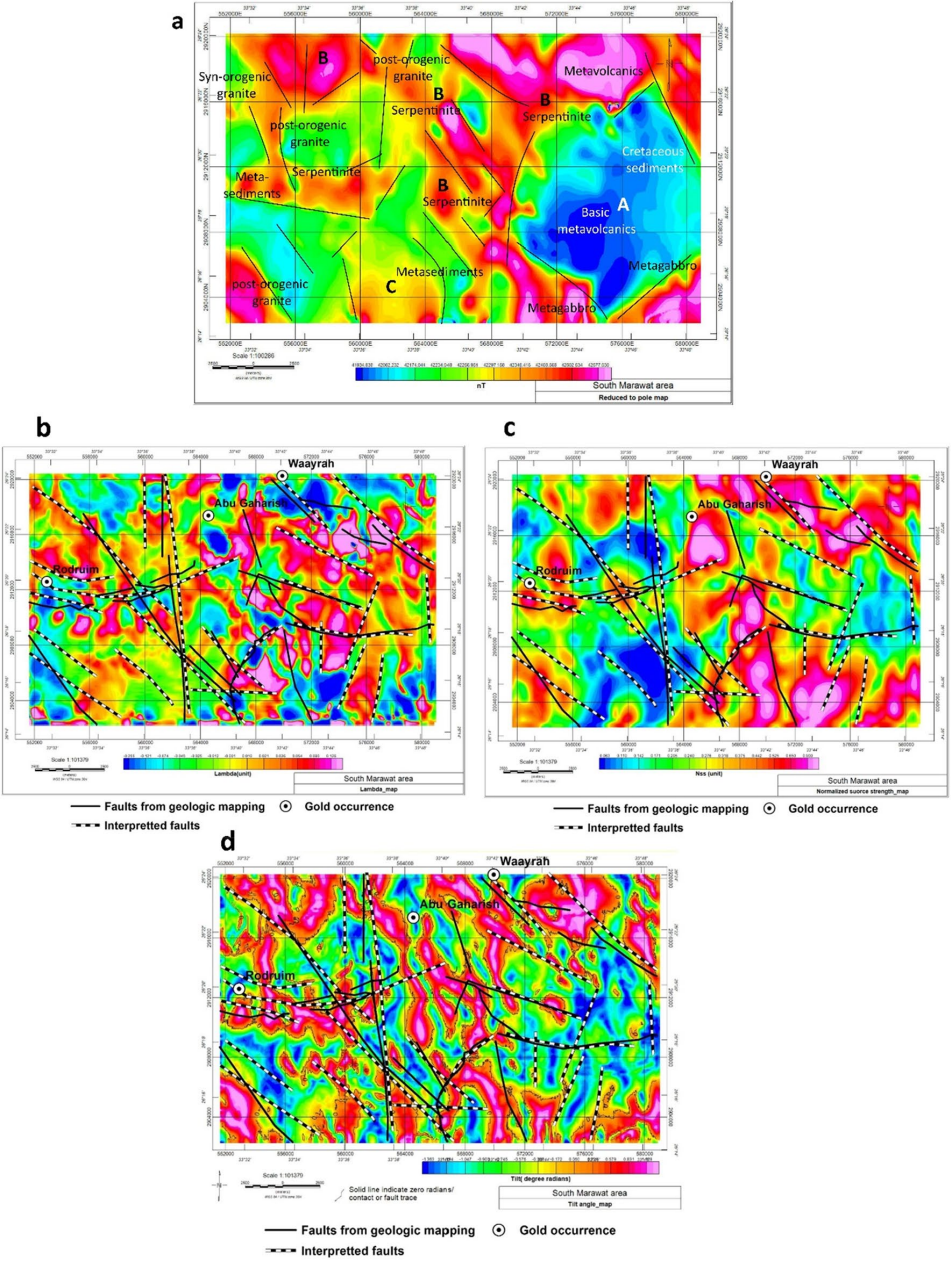

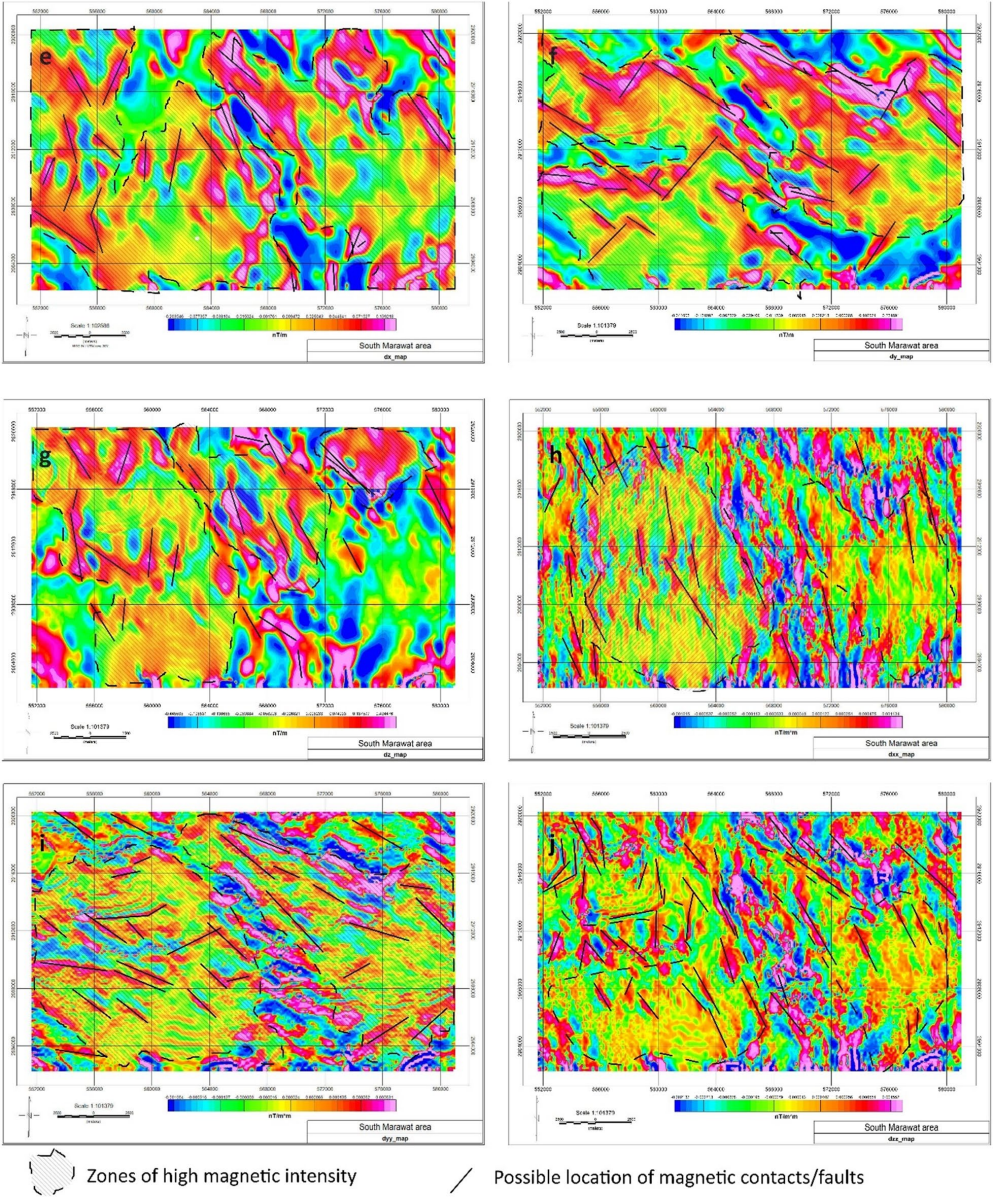
(**a**) Interpreted reduced to pole anomaly map for the study area, (**b**) Faults from geological mapping and faults interpreted from aeromagnetic data posted on **λ**_2_ map, (**c**) Faults from geological mapping and faults interpreted from aeromagnetic data posted on NSS or ${\varvec{\mu}}$ map, and (**d**) Faults from geological mapping and faults interpreted from aeromagnetic data posted on Tilt angle map. Structural data are projected using ArcGis-10.3 software, NSS is produced using Modelvision V17^68^ Software, while RTP and Tilt maps are produced using Oasis montage V 8.4 software^69^. Derivative analyses (**e**) dx, (**f**) dy, (**g**) dz, (**h**) dxx, (**i**) dyy, and (**j**) dzz. Zones of high magnetic intensity and possible contacts are posted on all maps. The above derivative maps are produced using Oasis montage V 8.4 software^69^.

Magnetically, the qualitative interpretation of the RTP map (Fig. 5a) shows that it is characterized by a remarkably high relief (~ 700 nT). A relatively low magnetic anomaly is observed to the east of the study area (A) corresponding to the cretaceous sediments and basic metavolcanics (< 42000nT), while the serpentine is characterized by a high magnetic signature (B) (> 42502 nT). An intermediate anomaly (C) corresponds to metasediments and post-orogenic granite is recorded in the southern and central parts of the area. qualitatively, different magnetic contacts can be observed and marked as black lineaments. These contacts separate the magnetic map into different zones. In comparing these signatures, the different rock units can be differentiated. Further analysis with derivatives (Fig. 5e–j) shows dominant magnetic trend directions are the NW–SE, NE-SW, and WNW-oriented faults sinistrally offset-thrust contacts that are dominant in the eastern part of the study area and related to oriented syncline affecting the Cretaceous Nubian sandstone. The normalized source strength transformation map ${\varvec{\mu}}$, together with the two complementary maps, ${{\varvec{\lambda}}}_{2}$ and $\boldsymbol{\varphi }$ is calculated. Faults from geological mapping and faults interpreted from aeromagnetic data posted on **λ**_2_, NSS or **μ**, and tilt angle maps (Fig. 5b,c,d). There is a remarkable similarity between the mapped and interpreted faults. Most faults are of NW to NNW trends. Based on the anomaly distribution, a few interpreted NE-SW faults from the aeromagnetic data are not recorded on the surface geological mapping. They are most probably representing subsurface structures.

### Aeromagnetic signature of different rock units

**Figure 6:**
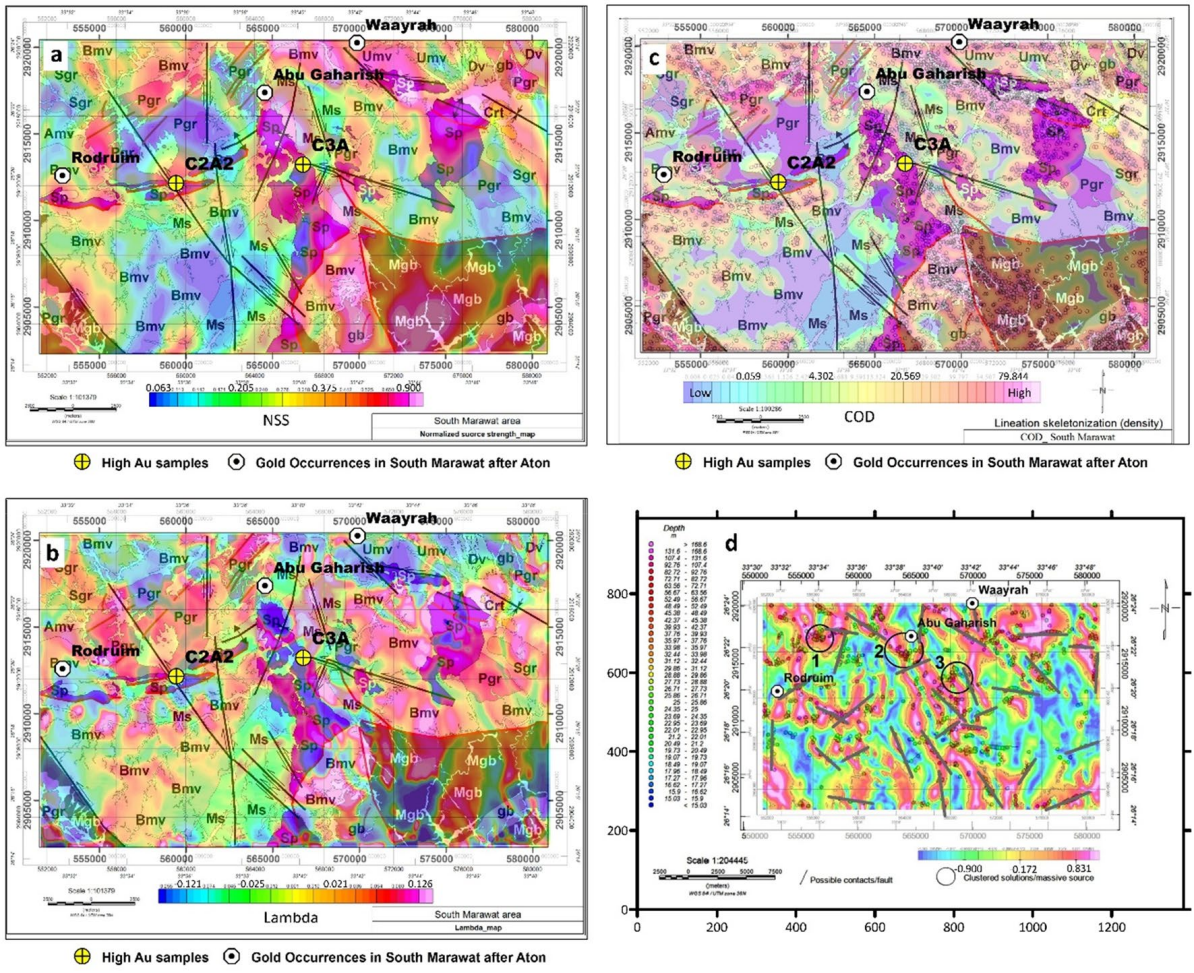
(**a**) The geology of the study area as posted on the NSS map. (**b**) The geology of the study area is posted on **λ**_**2**_ map., (**c**) The geology of the study area is posted on the COD map and (**d**) Faults are interpreted from the distribution of the Euler depth points posted on the tilt-angle map in which the zero-contour is illustrated. White circles are locations of gold occurrences. *Sp* serpentinite, *Mgb* metagabbro. *gb* fresh gabbro, *Bmv* Basic metavolcanics, *Umv* undifferentiated metavolcanics, *Fmw* intermediate to acidic metavolcanics, *Ms* metasediments, *Pgr* post orogenic granite, *Sgr* syn orogenic granite, *Dv* Dokhan volcanic, *Crt* Cretaceous sediments. White circles are locations of gold occurrences, and high Au sample locations are posted as yellow circles. Geological data are projected using ArcGis-10.3 software. **λ**_**2**_ is produced using Modelvision V17^68^, while for COD, Euler depths, and tilt maps, the Oasis montage V 8.4 software^69^ is used.

For comparison between the geology of the area and the resulting magnetic transformed anomalies **µ (**NSS**)** and** λ**_**2**_ maps, the distribution of the different rock units is projected on the maps (Fig. 6a,b). Although **λ**_**2**_ is an intermediate eigenvalue, remarkable features are observed. Three gold occurrences are cited by^70^, Waayarah occurrence in the undifferentiated metavolcanics, Abu Gaharish in the post-orogenic granite close to the contact against the metasediments, and Rodruim in the Basic metavolcanics. The locations of these occurrences are shown in Fig. 6a,b.

The integrated magnetic and geologic analysis shows that the three known gold occurrences are associated with intermediate to high anomalies in the NSS map (Fig. 6a) and low to intermediate anomalies in the **λ**_**2**_ data (Fig. 6b). The serpentinite generally shows high to very high anomaly areas in both the NSS and **λ**_**2**_ data, except in some parts attains intermediate to low anomalies due to its alteration to talc carbonate rocks, particularly in the western parts of the area. The metagabbro and fresh gabbro are of high to a very high anomaly in both the NSS and **λ**_**2**_ map except in altered parts along fault planes they show low anomaly.

The metasediments, on the other hand, show low to intermediate anomalies in the NSS data and medium to high anomalies in the **λ**_**2**_ map, and the basic metavolcanic and the undifferentiated metavolcanic attain variable magnetic signatures. In the NSS map, they have low to intermediate anomalies in the central and eastern parts of the area and higher values in the northern parts. In the **λ**_**2**_ map, they attain intermediate to very high anomalies at the central and eastern parts of the area and low values at the northern parts. The intermediate to acidic metavolcanic also gives variable magnetic signatures; it shows a low to an intermediate anomaly in the NSS data and a high anomaly in the **λ**_**2**_ map. the syn orogenic granite generally is of low to an intermediate magnetic anomaly in the NSS map and a high anomaly in the **λ**_**2**_ data. The post-orogenic granite generally attains low anomaly in the NSS data except for the northern parts of the NW intrusion, which shows high anomalies, In the **λ**_**2**_ map, it is generally of high anomaly, except for the northern parts of the NW intrusion, which shows a low anomaly. The Dokhan volcanic attain high magnetic anomaly in the NSS data and low anomaly in the **λ**_**2**_ map. The Cretaceous sediments generally show a high anomaly in the NSS and **λ**_**2**_ data. Two altered serpentinite rock samples of high gold content are found in this area (C2A2=2.2 ppm Au; C3A=1.7 ppm Au). These samples lie in intermediate to a high anomaly in the NSS map (contact zones), and in intermediate to a low anomaly in the **λ**_**2**_ map.

Generally, the variation in the magnetic signature within the same rock unit in the same magnetic data type is due to variations in the mineralogical composition within this unit (as confirmed by the field geology). In serpentinite, this variation is due to the alteration to talc carbonate rocks.

The CET grid analysis results are shown in Fig. 6. In Fig. 6c, the area's geology is projected on the contact occurrence density (COD) map, which shows the density of the fractures/faults in the area. The clusters in the COD map are strongly associated with all serpentinite exposures, most parts of the metagabbro, the northern exposures of the Basic, and undifferentiated metavolcanic. While it is weakly associated with the metasediments, syn orogenic and post-orogenic granites, central and southern exposures of the Basic metavolcanics, intermediate to acidic metavolcanics, Dokhan volcanic, and Cretaceous sediments. The two samples of high Au content (C2A2=2.2 ppm Au; C3A=1.7 ppm Au) lie within less fractured zones. Similarly, the Euler convolution depth solutions and interpreted faults/lineaments are posted on the tilt-angle map (Fig. 6d).

Analysis of Fig. 6d shows that many Euler depth solutions lie directly on the tilt-angle map's zero-contour, indicating that these solutions are associated with subsurface contacts or faults. The northern gold occurrence^70^ lies on the zero contours of the tilt-angle map, while the other two occurrences lie close to the zero contours, which indicates that the gold occurrence is directly associated with subsurface faulting and shearing. Additionally, the faults interpreted from the Euler points distribution are matching with the zero-contour of the tilt-angle map in many cases, which indicates that these interpreted faults do exist in the subsurface. Moreover, the clustered solution (number 1, Fig. 6d) lies along the lithologic contact between the post-orogenic granite and the Basic metavolcanics, while clustered solution (number 2) lies along the lithologic contact between the serpentinite and post-orogenic granite, and finally, clustered solution (number 3) lie along the fault contact between the metasediments and the post-orogenic granite.

### Land magnetic

A detailed land survey was performed along chosen three profiles through the target rock units for additional magnetic tomographic detailed inversion. As illustrated in Fig. 3a, a total of 178 stations were gathered along three long profiles (P11, P22, and P31).

The geologic interpretation of magnetic profiles is based on the data from geological mapping and structural studies on the area. Three gold occurrences are cited by^70^ within the study area Rodruim, Waayrah, and Abu Gaharish. The latter location is along the start of profile P22, (Fig. 3a). The distribution of these profiles is shown in Fig. 3a.

**Figure 7:**
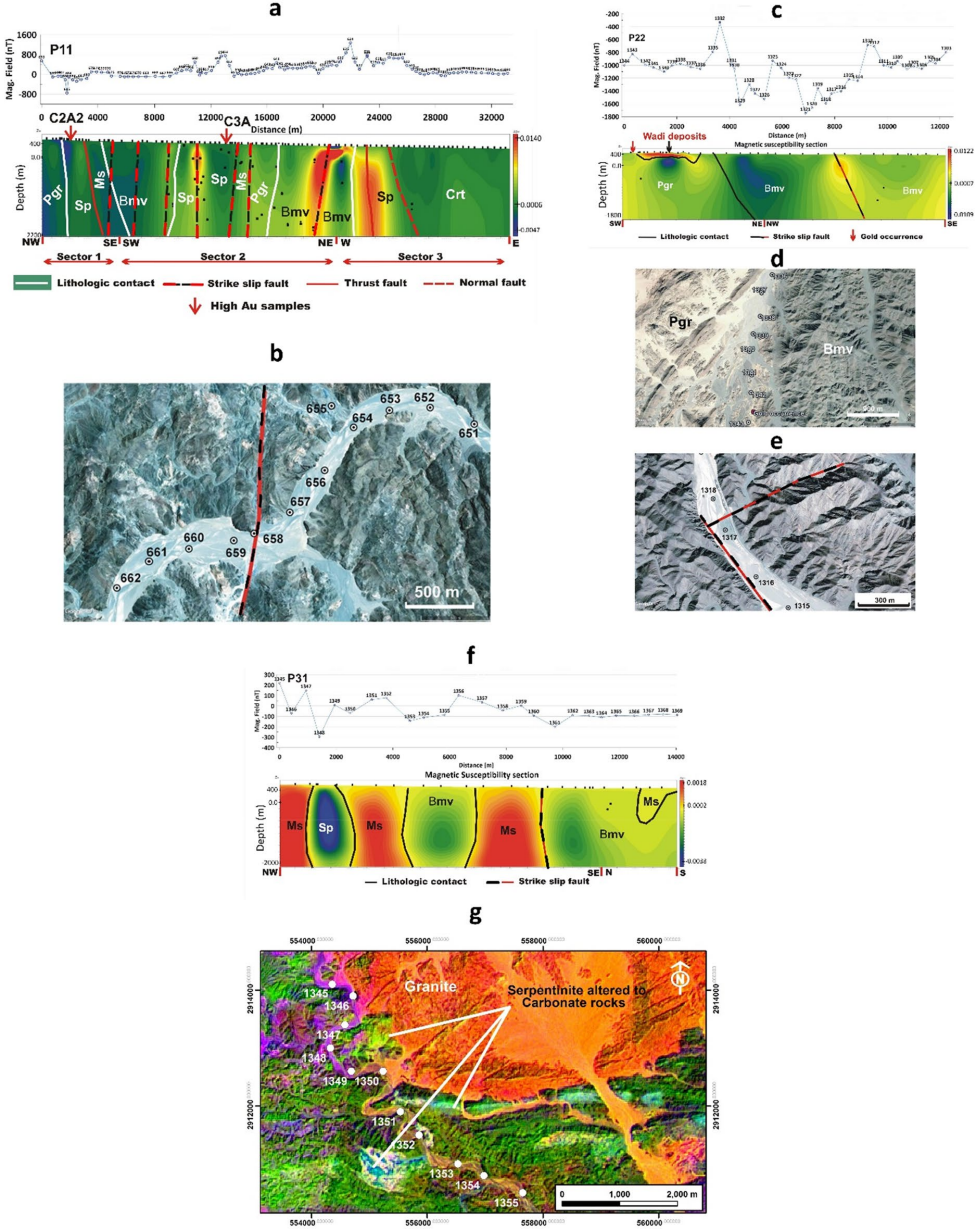
(**a**) Geologic interpretation of the magnetic field of profile P11, (**b**) High-resolution Google Earth satellite image showing a minor strike-slip fault cutting across profile P11 at station 658 in the serpentinite, (**c**) Geologic interpretation of the magnetic field of profile P22, (**d**) High-resolution satellite Google Earth image showing the wadi deposits of high magnetic anomaly between stations 1336 and 1343 (P22) within the Pgr rock unit, and (**e**) High-resolution Google Earth satellite image showing minor strike-slip faults cutting across profile P22 at station 1317 in the basic metavolcanics. Notice the dyke swarm across the granite and the dark grey colour of the wadi sediments, (**f**) Geologic interpretation of the magnetic field of profile P31, and (**g**) ASTER satellite image (ratio 2/1, 3/4, 4/7 for RGB, data downloaded from https://earthexplorer.usgs.gov, processed by Envi-5.3 software) for the NW part of profile P31 showing the alteration of the serpentinite to carbonate rocks between stations 1347 and 1349. *Sp* serpentinite, *Ms* metasediments, *Bmv* basic metavolcanics, *Bmv* basic metavolcanics, *Pgr* post-orogenic granite. *Pgr* post-orogenic granite. *Crt* Cretaceous sediments. White circles are traverse stations, Profile modeling is performed using ZondMag2D software^71^.

Profile P11 runs in three sectors trending NW-SE, SW-NE, and W-N., namely sectors 1, 2, and 3 (Fig. 7). The total length of the profile is about 32 km (Fig. 3a). The geologic interpretation (Fig. 7a) shows at the start of the profile (sector 1) the post-orogenic granite intruding the serpentinite and basic metavolcanics. The contact between the latter two units is a thrust contact. While in sector 2, the serpentinite, metasediments, and post-orogenic granite are in structural contact along N-S-oriented strike-slip faults.

The un-mappable minor strike-slip fault occurs at station 658 (Fig. 7b) within the serpentinite showing a high magnetic anomaly. At the end of this sector, the basic metavolcanics are affected by a strike-slip fault that shows a remarkably strong anomaly. In the third sector, the basic metavolcanics are in lithologic contact with the serpentinite. The serpentinite is affected by a thrust fault and is against the Cretaceous sediments along a normal fault.

The metasediments, basic metavolcanics, post-orogenic granite, and Cretaceous sediments show low magnetic anomaly, while the serpentinite is of a high anomaly in sector 3. The latter attains low anomaly in sectors 1 and 2 due to alteration to talc carbonates as indicated in the field and satellite images. Geochemical analysis shows that two altered serpentinite rock samples of high gold content almost lie along this profile (CcA2=2.2 ppm Au; C3A=1.7 ppm Au). These samples (C2A2; C3A) are located within the altered serpentinite rocks of low magnetic anomaly.

Profile 22 extends along two sectors trending SW-NE, and NW-SE from the start of the traverse. The profile length is about 12 km (Fig. 3a). The traverse runs through the post-orogenic granite and basic metavolcanics which are in lithologic contact and show low to medium magnetic anomaly (Fig. 7c). This profile shows that the Abu Gaharish gold occurrences cited by^70^, lie at the start of the traverse in the post-orogenic granite showing low surface magnetic anomaly and low inverted subsurface magnetic susceptibilities (Green colour) (Fig. 7c) and the wadi deposits within the post-orogenic granite close to the contact against the basic metavolcanics reflect a high magnetic anomaly. These deposits have a dark grey colour in the field and on the satellite images (Fig. 7d). This anomaly is most probably due to the high concentration of iron oxides, and a minor strike-slip fault within the basic metavolcanics (Fig. 7e) shows a high anomaly. This high anomaly musks the expected low surface magnetic anomaly. However, the magnetic susceptibility section shows that a low susceptibility target exists at the southwestern side of the profile (Abu Gaharish prospect). According to^72^, A 5 km extension of structurally controlled gold mineralization was confirmed by the assaying of more than a hundred grab and channel samples, which revealed considerable gold values (up to 27 ppm Au). Among his results of surface channel sampling are 31.2 m @ 1.04 g/t Au and 3.6 m @ 11.05 g/t Au. Moreover, according to ground penetrating radar, blind mineralization may occur beneath wadi alluvium (https://www.atonresources.com/). So, a very high probability of related fault zones affecting Abu Gaharish is present.

In profile P31, a total of 14 km of magnetic data are measured. Two sectors trending NW-SE and N-S from the start of the traverse, can be identified (Fig. 3a). The traverse cuts through the serpentinite, metasediments, and basic metavolcanics (Fig. 7f). The metasediments generally show high magnetic anomaly and are juxtaposed along a strike-slip fault against the basic metavolcanics at the eastern parts of the traverse. The serpentinite displays remarkably low anomaly. This is due to the alteration to talc carbonates as indicated in the ASTER data (Fig. 7g).

## Conclusions

The integration between the geological, geochemical, and aeromagnetic data was done through data overlay in ArcGIS environment. The integration between the geological, geochemical, and ground magnetic data was done by projecting the magnetic profile path on the geological map and then comparing the lithologic contacts and structural lines of the geologic map (together with sample locations and field geologic results) with the ground magnetic stations of the profile. Accordingly, the contacts and faults are placed in their exact position at the top of the profile and then extended through the subsurface as expected from the geological mapping and the distribution of the magnetic susceptibilities resulting from the tomographic inversion of these profiles.

Based on the integrated investigation and comparison between the results from different techniques including geologic site investigation, and different magnetometric filtering, the following conclusions are drawn:

- The gold occurrences (Abu Gaharish) cited by^70^ lie in the post-orogenic granite (P22) showing low magnetic anomaly.
- Gold was detected in the soapstone samples (1.7–2.2 ppm) as evidenced by the geochemical analysis, this is associated with low magnetic anomalies due to the associated carbonate’s low magnetic susceptibility. This constitutes a future possibility of gold potentiality in a possible large-scale gold mining (LSGM) locality in the South Abu Marawat area.
- The two samples of high gold content (C2A2 = 2.2 ppm Au; C3A = 1.7 ppm Au) lie within the contact occurrence density peaks, i.e. gold mineralization in south Abu Marawat is generally highly associated with fractures and/or faulted zones with low magnetic susceptibility zones.
- Some strike-slip faults show high magnetic anomaly, due to shearing along fault planes and probable concentration of iron oxides by hydrothermal solutions.
- The wadi deposits within the post-orogenic granite at the start of profile P22 close to the contact against the basic metavolcanics reflect a high magnetic anomaly. These deposits are dark grey in the field and on satellite images. This anomaly is most probably due to the high concentration of iron oxides.
- The metasediments show medium to high magnetic anomaly, while the serpentinite and basic metavolcanics attain low anomaly probably due to their partial alteration to carbonate rocks. The post-orogenic granite and Cretaceous sediments are always of low anomaly.
- The proposed strategy in exploring gold mineralization zones using integrated COD, NSS, tilt magnetic maps, and magnetic tomographic modelling together with RS and geochemical evidence, could be used as a criterion in similar areas of complex structures for gold mineralizations.
- As a conclusion, the gold mineralization in the study area is linked to the altered ultramafic zones that are associated with faulting and shearing and characterized by low subsurface magnetic susceptibility.

## Data Availability

The data that support the findings of this study are available from the Science, Technology & Innovation funding Authority (STDF) (under project ID: 26277), but restrictions apply to the availability of these data, which were used under license for the current study, and so are not publicly available. Data are however available from Prof. Mohamed Mostafa Gobashy as the Co. PI of the project (gobashy@CU.edu.eg) upon reasonable request and with permission of (STDF).
